# Geriatric Care Models

**DOI:** 10.3390/geriatrics6010006

**Published:** 2021-01-12

**Authors:** James S. Powers

**Affiliations:** 1 The Department of Veterans Affairs Tennessee Valley Healthcare System Geriatrics Research, Education, and Clinical Center, Nashville, TN 37212, USA; james.powers@vumc.org; 2 The Center for Quality Aging, Vanderbilt University School of Medicine, Nashville, TN 37232, USA

This Special Issue on geriatric care models features 18 papers highlighting the evolving nature of healthcare delivery and the leadership and quality enhancement research provided by geriatric care models [1]. These papers extend our knowledge of pre-implementation, implementation, and sustainment for these innovative models for home care, primary care, emergency medicine, nursing home care, transitions of care, and acute care for older adults.

Home-based primary care is embraced by health systems that reward value over volume and target the most complex patients, providing services such as hospital at home, and improving the quality of life for home-limited patients and their caregivers [2].

Innovative programs to care for frail older adults in the community include the Case–Finding for Complex Chronic Conditions in Seniors 75+ Program—a proactive approach to identify frail elderly people at highest risk of poor outcomes that targets multidisciplinary interventions to maintain health and well-being [3]. Geriatric Patient-Aligned Care Teams (GeriPACTs) have been developed by the Department of Veterans Affairs (VA) as patient-centered medical homes for older adults, providing high-quality coordinated care [4]. GeriPACTs employ a range of staffing and clinical structures that are adapted to local needs. Clinical video telehealth (CVT) is also being employed by the VA to improve access for rural residents [5] and for consultative services, including dementia care and caregiver support [6]. Telehealth appears to be well accepted by patients and effective in optimizing geriatric care and obtaining community-based long-term care services and support (LTSS).

The Emergency Department (ED) is uniquely positioned to improve the care of older adults. A dedicated Geriatric ED can replicate an Acute Care for Elders model of interdisciplinary care to provide comprehensive care plans for vulnerable older adults at risk of morbidity, ED revisits, and potentially avoidable hospital admissions [7].

Nursing home residents can receive access to specialty care delivered to nursing homes using video visit technology, as demonstrated by the Vet Connect Program [8]. Active case management, as described in the Return to Community Initiative (RTCI), holds promise as a successful model to assist individuals at risk of becoming long-stay nursing home residents to instead return to the community [9].

Suboptimal care transitions increase the risk of adverse events and result from poor care coordination among providers and healthcare facilities. Facilitation of a smooth and seamless transition relies on the abilities of skilled nursing facilities (SNFs) and primary care teams, as well as community agencies, to coordinate together in a patient-centered manner [10]. Geriatric syndromes and polypharmacy are common in older adults discharged to SNFs and increase 30-day readmission risk [11]. The continuity of care, interdisciplinary team, and advanced open access provided by GeriPACT can contribute to improved outcomes in 30-day all-cause readmissions [12].

Acute Care for Elders (ACE) Units show increased adherence to evidence-based geriatric processes, improve patient functional status at the time of hospital discharge, and reduce the length of stay and costs for older adults [13,14,15]. Improved hospital care of older adults can also be realized by collaborative orthopedic co-management services [16] and implementation of early hospital mobility programs to maximize functional status at the time of discharge [17].

Comprehensive geriatric assessment has become a fundamental part of clinical geriatric care. Geriatric evaluation and management have evolved and are now occurring in long-term care, homes, and outpatient settings [18]. Availability of geriatric resources and trained personnel across the continuum of care is critical to successful implementation and sustainment of these innovative models of care for older adults that have been found to be successful in many varied healthcare systems worldwide.
